# Systematic challenges and opportunities in insect monitoring: a Global South perspective

**DOI:** 10.1098/rstb.2023.0102

**Published:** 2024-06-24

**Authors:** Melissa Sánchez Herrera, Dimitri Forero, Adolfo Ricardo Calor, Gustavo Q. Romero, Muzafar Riyaz, Marcos Callisto, Fabio de Oliveira Roque, Araseli Elme-Tumpay, M. Kawsar Khan, Ana Paula Justino de Faria, Mateus Marques Pires, Carlos Augusto Silva de Azevêdo, Leandro Juen, Usman Zakka, Akeweta Emmanuel Samaila, Suwaiba Hussaini, Kehinde Kemabonta, Rhainer Guillermo-Ferreira, Blanca Ríos-Touma, Gyanpriya Maharaj

**Affiliations:** ^1^ Department of Museum Research and Collections, University of Alabama Museums, Tuscaloosa, AL 35487, USA; ^2^ Division of Invertebrate Zoology, American Museum of Natural History, New York, NY 10024, USA; ^3^ Laboratorio de Zoología y Ecología Acuática (LAZOEA), Biological Sciences Department, Universidad de los Andes, Bogotá, 111711, Colombia; ^4^ Instituto de Ciencias Naturales, Universidad Nacional de Colombia, Bogotá, 11132, Colombia; ^5^ Instituto de Biologia, Laboratório de Entomologia Aquática, Universidade Federal da Bahia, Salvador, 40000-000, Brazil; ^6^ Laboratório de Interações Multitróficas e Biodiversidade, Departamento de Biologia Animal, Instituto de Biologia, Universidade Estadual de Campinas (UNICAMP), CP 6109, Campinas-SP, CEP 13083-970, Brazil; ^7^ St Xavier's College, Palayamkottai, Tirunelveli, Tamil Nadu, CEP: 40170-115 7 – 627002, India; ^8^ Instituto de Ciências Biológicas, Universidade Federal de Minas Gerais, Genética, Ecologia e Evolução, Pampulha, Belo Horizonte - MG, 31270-901, Brazil; ^9^ Departamento de Biología, Universidade Federal de Mato Grosso do Sul, Ciudade Universitaria, Pioneiros, Campo Grande, MS, 79070-900, Brazil; ^10^ Centre for Tropical Environmental and Sustainability Science and College of Science and Engineering, James Cook University, Douglas, Cairns, 4811, Queensland, Australia; ^11^ Laboratorio de Biodiversidad y Genética Ambiental (BioGeA), Universidad Nacional de Avellaneda, Mario Bravo 1460, CP1870 Piñeyro, Avellaneda, Buenos Aires, Argentina; ^12^ Colección Entomológica, Universidad Nacional de San Antonio Abad del Cusco, Gabinete C-338, Pabellón C, Ciudad Universitaria de Perayoc, Cusco, 08003, Peru; ^13^ Department of Biology, Chemistry and Pharmacy, Freie Universität Berlin, Berlin, 14195, Germany; ^14^ Instituto de Ciências Biológicas, Universidade Estadual do Piauí, Rua João Cabral - Matinha, Teresina - PI, 64018-030, Brazil; ^15^ Laboratory of Ecology and Conservation of Aquatic Ecosystems, Universidade do Vale do Taquari - UNIVATES, Lajeado, RS, 95914-014 Brazil; ^16^ Departamento de Biología, Universidade Estadual do Maranhão, Programa em Biodiversidade, Ambiente e Saúde, 65.055-310, Brazil; ^17^ Instituto de Ciências Biológicas, Universidade Federal do Pará, UFPA, Belém - PA, 66077-830, Brazil; ^18^ Department of Crop & Soil Science, University of Port Harcourt, Port Harcourt 500272, Nigeria; ^19^ Department of Agronomy, Federal University of Kashere: Kashere, P.M.B. 0182, Gombe State, Nigeria; ^20^ Department of Biological Sciences, Abubakar Tafawa Balewa University, Bauchi, 740272, Nigeria; ^21^ Department of Zoology, University of Lagos: Akoka, Lagos, 100213, Nigeria; ^22^ Centro de Pesquisas em Entomologia e Biologia Experimental, Universidade Federal do Triangulo Mineiro (UFTM), Uberaba - MG, 38061-500, Brazil; ^23^ Grupo de Investigación en Biodiversidad, Medio Ambiente y Salud (BIOMAS), Universidad de Las Américas, Campus UDLAPARK, Quito, Ecuador 170513; ^24^ University of Guyana, Centre for the Study of Biological Diversity, Georgetown, Guyana

**Keywords:** biodiversity conservation, insect biomonitoring, socio-economic challenges, language inclusivity, technological collaborations

## Abstract

Insect monitoring is pivotal for assessing biodiversity and informing conservation strategies. This study delves into the complex realm of insect monitoring in the Global South—world developing and least-developed countries as identified by the United Nations Conference on Trade and Development—highlighting challenges and proposing strategic solutions. An analysis of publications from 1990 to 2024 reveals an imbalance in research contributions between the Global North and South, highlighting disparities in entomological research and the scarcity of taxonomic expertise in the Global South. We discuss the socio-economic factors that exacerbate the issues, including funding disparities, challenges in collaboration, infrastructure deficits, information technology obstacles and the impact of local currency devaluation. In addition, we emphasize the crucial role of environmental factors in shaping insect diversity, particularly in tropical regions facing multiple challenges including climate change, urbanization, pollution and various anthropogenic activities. We also stress the need for entomologists to advocate for ecosystem services provided by insects in addressing environmental issues. To enhance monitoring capacity, we propose strategies such as community engagement, outreach programmes and cultural activities to instill biodiversity appreciation. Further, language inclusivity and social media use are emphasized for effective communication. More collaborations with Global North counterparts, particularly in areas of molecular biology and remote sensing, are suggested for technological advancements. In conclusion, advocating for these strategies—global collaborations, a diverse entomological community and the integration of transverse disciplines—aims to address challenges and foster inclusive, sustainable insect monitoring in the Global South, contributing significantly to biodiversity conservation and overall ecosystem health.

This article is part of the theme issue ‘Towards a toolkit for global insect biodiversity monitoring’.

## 1. Introduction

Insects play critical roles in shaping habitat dynamics [1], providing a broad range of ecosystem services [2,3], serving as integral components of the food webs and pollinating major food sources across the globe [4–7]. As with biodiversity worldwide, declining insect populations are of global concern as insect communities are indispensable for global sustainability and human development [4,5]. This decline is particularly pronounced in the tropics, home to more than 3.9 million of the approximately 5.5 million insect species in the world [4]. Furthermore, while insects constitute the predominant segment of macroscopic terrestrial animal life and insect pollinators contribute to more than one-third of global crop production [6], our comprehension of insect responses to anthropogenic threats remains poor. Therefore, to ensure that insects are protected and conserved, there is a need to understand past population trends in response to stressors, and for a greater understanding of insect biology and ecology [5] and how insect populations are currently changing. As such, there is an urgent imperative for sustained, comprehensive monitoring, especially in substantially under-sampled regions, such as the Global South^1^, to add to the paucity of global invertebrate conservation literature [4,8], and to ascertain the applicability of findings from temperate studies, which currently provide robust empirical support for these alarming declines in insect populations [8–10].

Given the potential heightened sensitivity of insects to climate change, it is important to allocate increased attention to their study [4,8]. The identification of key functional arthropod taxa in tropical terrestrial and aquatic habitats through functional traits provides a basis for developing long-term monitoring programmes [2]. These programmes should prioritize collaboration with local researchers and be adaptable to the specific challenges faced in the Global South, ensuring inclusivity and sustainability [9,11,12]. By advocating for tailored approaches and addressing these challenges, we can foster a more equitable distribution of knowledge and contribute to conserving vulnerable species and habitats in these regions [10,12].

Recently, Lamarre *et al*. [10] and Slade & Ong [12] underscored the following key challenges in insect biomonitoring within tropical regions. Among these, a primary obstacle is the extraordinary diversity of insect species in tropical ecosystems, presenting a formidable challenge for their comprehensive identification and monitoring. Compounding this, the scarcity of taxonomic expertise [13] and resources in many tropical countries further impedes the identification and monitoring of insect species [14]. The logistical complexities associated with working in remote and challenging-to-reach tropical ecosystems, such as forests in outlying mostly inaccessible areas, add a layer of difficulty to insect monitoring initiatives [14]. Additionally, establishing and maintaining long-term, continuous monitoring programmes can be resource-intensive, demanding sustained funding commitments that are often unable to be met by local budgetary allocations. Lastly, the active involvement of local communities and stakeholders becomes paramount for garnering support for conservation endeavours and ensuring the enduring sustainability of monitoring programmes through conservation policy [15].

Considering these challenges, our review paper aims to give voice to local researchers in the Global South grappling with the intricacies of insect monitoring research. This paper seeks to use their insights as a catalyst for fostering inclusive collaborations, shedding light on unique socio-economic cases that influence the development of standardized methodologies. Moreover, we aim to provide viable opportunities that resonate with the perspectives of local researchers and communities, facilitating the creation of localized biomonitoring programmes. Through a series of structured interviews and discussions among Global South experts, we endeavour to uncover region-specific concerns and potential solutions (see box 1). The paper underscores the urgency of addressing these challenges and leveraging the opportunities to advance insect monitoring in the most diverse regions in the world and how these efforts can be supported worldwide.

Box 1. **Structured survey protocol.**

To comprehensively capture diverse perspectives on insect monitoring across the Global South, authors Dr M. Sánchez-Herrera and Dr K. Kemabonta coordinated a comprehensive survey within the entomological community, and all respondents actively participated as co-authors in crafting this review paper. The survey delved into key aspects, including prevalent insect monitoring methods, their efficacy in respective regions, encountered challenges, and the impact of environmental or socio-economic factors on monitoring initiatives. Participants were asked about necessary resources and support to fortify insect monitoring efforts, highlighting specific species or groups of concern, valuable research papers, access to unpublished data and engagement of local organizations in monitoring or conservation. Regulatory or policy issues were explored, along with suggestions for collaborations and insights into technological adoption challenges or opportunities. A total of 22 colleagues from diverse regions across the Global South shared their perspectives, enriching the depth and breadth of our collective vision presented in this paper.

The following 11 open-ended suggestions were explored using the Google Forms platform:

1. **Prevalent insect monitoring methods:** Exploring the effectiveness of methods used in different regions.
2. **Encountered challenges:** Identifying main limitations faced during insect monitoring activities.
3. **Impact of environmental or socio-economic factors:** Assessing how specific factors affect insect monitoring in various areas.
4. **Needed resources and support:** Gathering insights on what is required to enhance insect monitoring efforts.
5. **Concerns about specific insect species or groups:** Highlighting species or taxonomic groups of concern and the reasons behind the concerns.
6. **Valuable research papers:** Identifying peer-reviewed publications related to insect monitoring in the Global South.
7. **Access to unpublished data:** Exploring the availability of data or case studies that could contribute to understanding insect monitoring.
8. **Involvement of local organizations:** Sharing information about organizations, communities or initiatives actively engaged in insect monitoring or conservation.
9. **Regulatory or policy issues:** Addressing any issues that need attention or improvement concerning insect monitoring.
10. **Suggestions for collaborations:** Providing insights into potential collaborations or partnerships to enhance monitoring efforts.
11. **Challenges or opportunities related to technology adoption:** Highlighting specific technological challenges or opportunities for insect monitoring in different regions.

The figure below shows word clouds for the suggestions provided by the author participants.

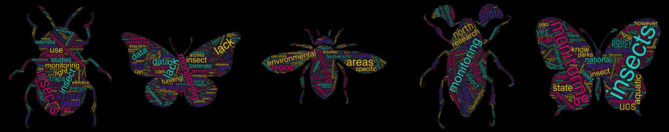

**Figure 1.**
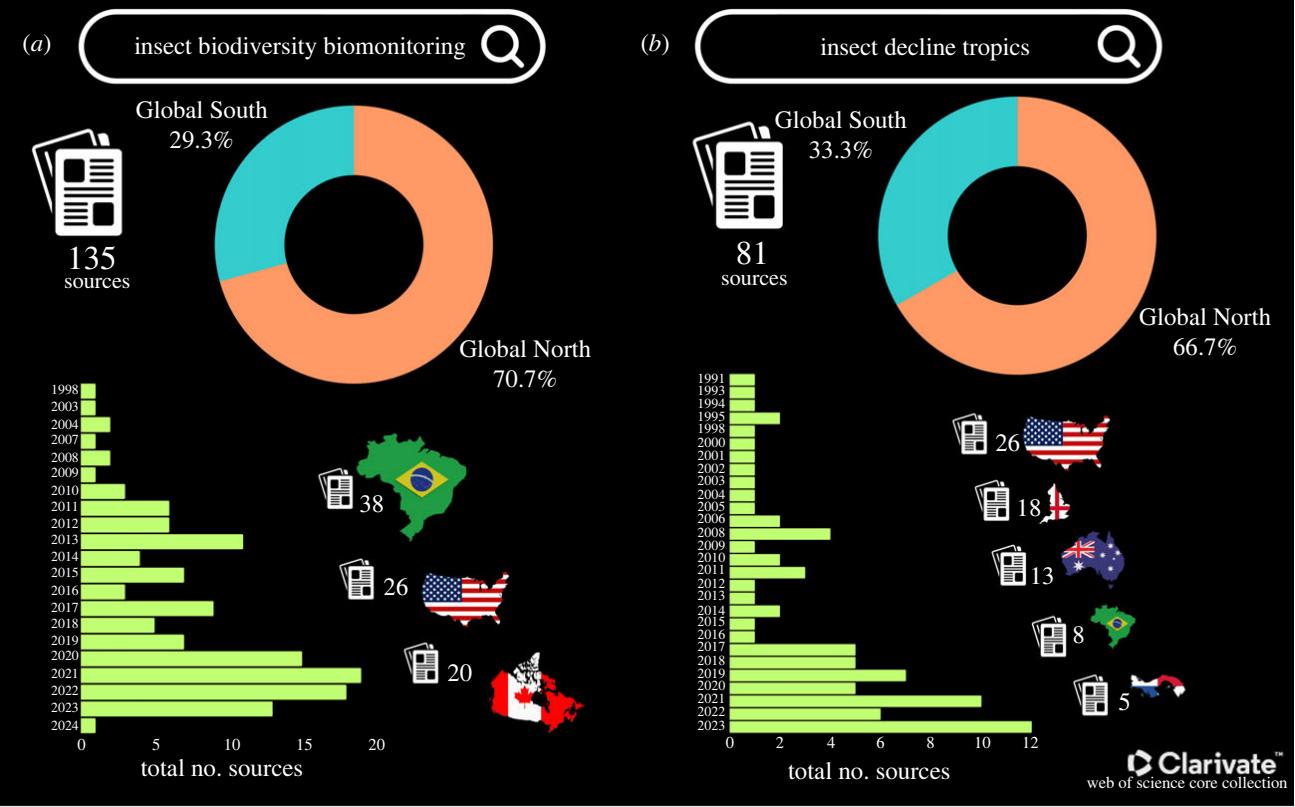
Discrepancies between the entomological publication records of the Global North and Global South based on keyword searches in the Web of Science Core Collection: (*a*) ‘insect biodiversity biomonitoring’ and (*b*) ‘insect decline tropics’. Each panel displays the total number of sources, pie charts representing the Global North and Global South, the number of publications by country, and the countries contributing the most to the recovered sources. (Data source: Web of Science Core Collection 1990–2024' electronic supplementary material, S1.)

**Box figure 1.** Wordle word clouds of the raw suggestions (1–5) of the Google Forms questionnaire.

## 2. Challenges in insect monitoring

In recent years, the field of insect biomonitoring within tropical regions has faced significant challenges [10,12]. Understanding these challenges is crucial for unravelling the complexities of insect dynamics, stemming from the extraordinary diversity of insect species within tropical ecosystems. This diversity poses a formidable challenge for comprehensive insect identification and monitoring. Examining the Web of Science Core Collection from 1990 to 2024, we extracted the number of sources for the following two combinations of keywords: (i) ‘insect biodiversity biomonitoring’ and (ii) ‘insect decline tropics' (see details in electronic supplementary material, S1). We obtained a total of 135 publications for the first combination and 80 publications for the second (figure 1; electronic supplementary material, S1). Interestingly, when we look at the country of origin, we see that approximately 70% of these sources are contributions from the Global North and only approximately 30% from the Global South (figure 1), which points out the imbalance in entomological research and publication. As discussed previously, the scarcity of taxonomic expertise and resources in many tropical countries, part of the Global South, compounds this difficulty, hindering the effective identification and monitoring of insect species. However, our perspective from the Global South highlights important and unique environmental and socio-economic issues that underlie the systemic challenges that limit resources and expertise for biodiversity biomonitoring, particularly of arthropods, in this region of the world.

### (a) Socio-economic factors

**Figure 2.**
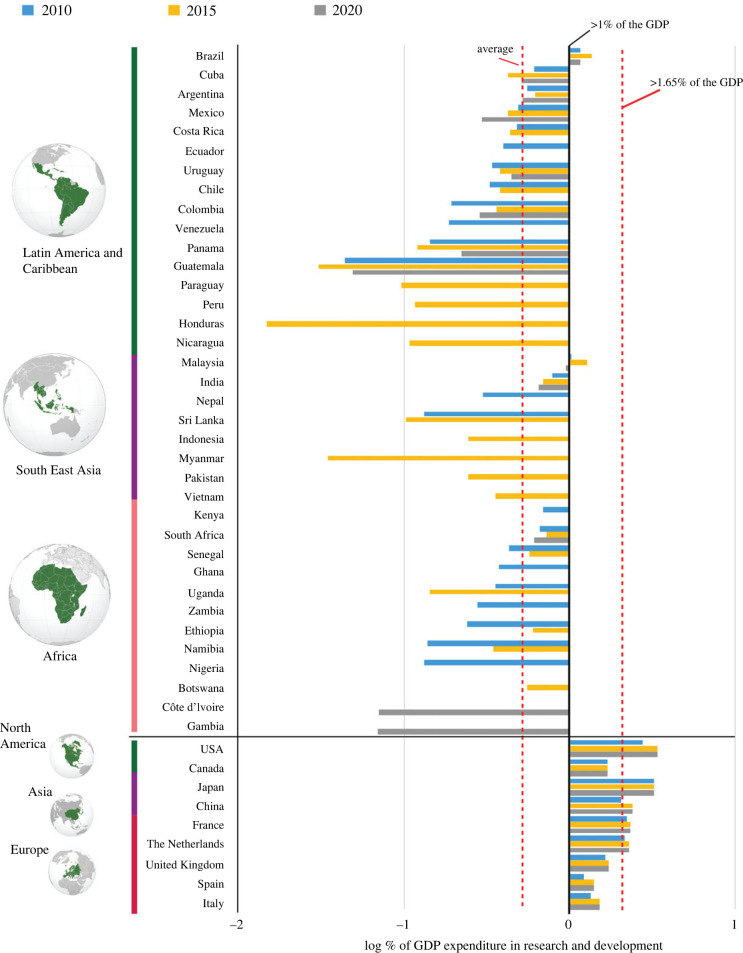
Log of GDP expenditure in research and development (R&D) worldwide (2010–2020). The graph compares regions categorized as Global South (Latin America and Caribbean, Southeast Asia and African countries) with Global North (North American, Asian and European countries). China is classified within the Global North owing to its strong economy. (Data source: World Bank 2023.)

One primary concern among researchers in the Global South is the inconsistent funding landscape, particularly from government sources, which is crucial for sustaining long-term biomonitoring efforts. The disparity in scientific investment across countries is stark, as evidenced by data on research and development expenditure as a percentage of gross domestic product (GDP) sourced from the United Nations Educational, Scientific and Cultural Organization (UNESCO) Institute for Statistics at the World Bank (figure 2). Tropical countries, on average, allocate approximately 3 percentage points less of their GDP to scientific endeavours compared with countries such as the United States, Japan, the UK and The Netherlands. Only Brazil and Malaysia among Global South nations invest more than 1% of their GDP in scientific and technological pursuits, with Brazil leading in publication records related to insect monitoring (figure 1). However, lack of continuous data on research investments in some regions, for example Indomalaya and Africa, highlights the challenge in obtaining comprehensive insights. While countries like India and South Africa provide significant data, many others lack transparency or face limitations in data availability, especially during disruptive events like the pandemic, which saw a significant decrease in investment across the Global South (figure 2).

Moreover, socio-economic factors exacerbate the funding shortfall for insect-related research. In terms of GDP, developing countries, despite hosting a significant portion of the global biodiversity, often spend less than 50% of the global average expenditure on biodiversity conservation [16]. This financial constraint extends to academic institutions, where researchers often struggle to secure funding and access essential resources for their work. Unlike their counterparts in developing countries, researchers in the Global South face limited opportunities for private funding [17], or for government grants dedicated to support insect-related research [12]. For example, in many Caribbean countries, long-term arthropod vector monitoring and control, and training of personnel in vector taxonomy and biology, are fiscally unsustainable and therefore often require external support [18].

This reliance on external support further perpetuates inequalities in research capacity, with academics from the Global South competing for resources against better-equipped counterparts from the Global North. Existing collaboration models, while offering opportunities, often fail to address local research capacity-building needs and may perpetuate dependency rather that fostering sustainable growth. In countries in Asia and Africa these collaborations may promote sending samples abroad, ‘parachute or helicopter research’, where foreign researchers visit countries for short periods and then leave, or advocate for locals to collect and send samples abroad, and/or for field research sites run by external researchers, or lure top local researchers from existing national institutes and universities with offers of greater incentives [19].

At the national level, the funding deficit severely impacts research infrastructure and capacity-building initiatives, hindering access to essential equipment, technology and training opportunities [20,21]. This lack of resources is particularly evident in fields like taxonomy and natural history, essential for insect identification and conservation efforts in tropical regions [12,22]. Furthermore, inadequate investment in information technology infrastructure and data management systems hampers data collection, storage and sharing efforts, essential for effective insect biomonitoring. The absence of standardized protocols and incentives for data sharing (e.g. the Global Biodiversity Information Facility, GBIF) further exacerbates this challenge, limiting collaboration and hindering the advancement of collective knowledge [23,24]. Finally, currency devaluation adds another layer of complexity, affecting the purchasing power of research budgets and exacerbating financial constraints, especially compared with agencies in the Global North with stronger currencies (e.g. US dollar, Euro, pound Sterling).

In conclusion, addressing the multifaceted challenges faced by tropical regions in insect biomonitoring in tropical regions requires collaborative efforts, sustainable funding mechanisms, and strategic investments in education, research and technological infrastructure. Only through comprehensive approaches can these regions overcome socio-economic barriers and pave the way for effective and sustainable insect monitoring, crucial for biodiversity conservation in critical ecosystems.

### (b) Environmental factors

The prevailing environmental conditions significantly shape insect biomonitoring efforts worldwide, particularly in tropical regions facing various environmental challenges [20]. Climate change, alongside issues like urbanization [24], land use conversion [25], pollution and poverty [26,27], presents complex obstacles to biodiversity preservation. Tropical areas, home to the majority of global biodiversity, are crucial for carbon sequestration and habitat preservation [27]. As such, there is a growing emphasis on research and development expenditure in these regions, driven by international agendas like the sustainable development goals (SDGs).

Central and South America exemplify regions facing significant environmental pressures. In Central America, climate change exacerbates vulnerabilities, prompting initiatives for climate resilience. The focus here is on enhancing resilience to climate change through initiatives such as the Central America Commission for Environment and Development's Regional Environmental Framework Strategy 2020–2025 and collective initiatives within the Caribbean Community (CARICOM). Additionally, the Green Climate Fund supports projects like geothermal resource development in several Caribbean nations, while Guyana's Sovereign Wealth Fund aims to transform Bartica into a green town, highlighting the need for increased support for biodiversity research in the region [20,28]. South America, home to diverse ecosystems like the Amazon Basin and páramos, confronts threats from urbanization, mining, agriculture and deforestation [29]. In Latin America, the fastest urbanizing region of the world [30], where urban population has surpassed rural since the 1980s [24], inadequate urban planning and sanitation practices (common direct disposal of sewage into natural waterways in the area, [31]) pose significant risks to both human populations and ecosystems supporting rich insect diversity [32]. Colombia, despite overcoming internal conflict, faces challenges in biodiversity exploration. Post-peace treaty, the ColombiaBio (https://minciencias.gov.co/portafolio/colombia-bio) programme emerged, promoting biodiversity conservation, management and sustainable use. However, funding limitations due to COVID-19 and subsequent government changes hinder continuous biomonitoring observation. Deforestation within Colombian Protected Areas and buffer zones has also surged post-conflict, challenging assumptions about automatic biodiversity protection [33]. Conversely, Brazil has been active in climate diplomacy and has hosted several international summits on climate change. However, recent political ideologies in government relaxed environmental laws and regulations, which is a cause for concern given the string of ecological disasters that Brazil has experienced in recent years [28,34]. The UNESCO Science Report calls for greater environmental monitoring in Brazil, as severe environmental crises have occurred owing to insufficient monitoring and prevention systems [20].

In Southeast Asia, rapid urbanization and high energy demands heighten vulnerability to climate change [35]. Bhutan's commitment to environmental conservation, guided by the Gross National Happiness philosophy, offers a model for sustainable development [20]. By contrast, countries like Bangladesh prioritize technological solutions to mitigate environmental challenges, reflecting diverse approaches within the region [20]. Moreover, India has made significant strides in promoting clean energy and reducing carbon emissions, and the latest UNESCO science report suggests that the country is actively promoting sustainable development and addressing environmental challenges. In Africa, biodiversity loss poses significant challenges to rural livelihoods, particularly for women. Conservation efforts, such as improved land use practices and investment in smallholder farmers, are critical for addressing food security and preserving ecosystem services [36].

As entomologists within and outside the tropical region, we should advocate for the crucial ecosystem services that insects provide for the surveillance and monitoring of various environmental issues [1–3,7,37,38]. Our expertise and research play a pivotal role in understanding and addressing the complex interplay between insects, climate change and the overall health of ecosystems, emphasizing the need for continued support and collaboration in preserving the invaluable biodiversity within these extremely biodiverse regions.

### (c) Ongoing biomonitoring programmes and logistic complexities

**Figure 3.**
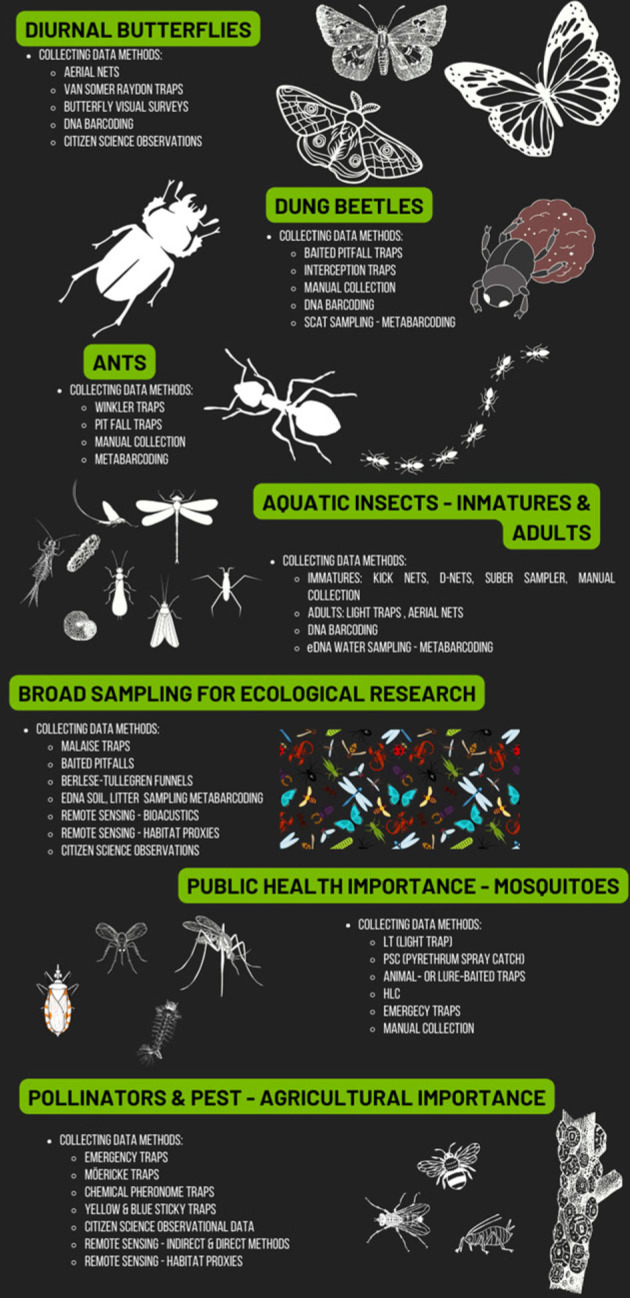
Focal taxa and effective collecting of data for monitoring insects in the tropical region. HLC, human landing catch. See [11,37,39,40].

In the domain of insect monitoring, diverse approaches and effectiveness are evident across different regions, as highlighted in figure 3 [39]. Focal groups, notably charismatic taxa like Lepidoptera (i.e. diurnal butterflies, skippers and moths) [32,41–46] and beetles (mostly dung beetles) [47–53], have been extensively studied, utilizing effective collection and observational survey methods that allow the establishment of standardized protocols replicable over time [54–56]. Substantial progress has also been achieved in freshwater monitoring programmes, with the immature stages of various aquatic orders playing a pivotal role [32,41,42,44,45,57–59]. Noteworthy biodiversity monitoring efforts in India, utilizing camera traps and portable photography devices like smartphone-macro lens setups, have significantly enhanced regional biodiversity documentation [60].

Despite these advancements, challenges persist in species identification, especially in insect groups for which there is limited expertise available [22,61,62]. While taxonomic knowledge has grown through international collaborations, this progress remains uneven across the vast diversity found in tropical ecosystems [63]. In Latin American countries like Panama and Brazil, biomonitoring programmes have seen an increase, driven by strong local taxonomical expertise and long-term collaborative initiatives like the Forest Global Earth Observatory (ForestGEO) programme run by the Smithsonian Tropical Research Institute [64]. For the Brazilian case, state organizations like the Compahia Ambiental do Estado de São Paulo (CETESB) are sponsoring a macroinvertebrate monitoring programmes. Other federal units like the Chico Mendes Institute for Biodiversity Conservation (ICMBio) is implementing the MONITORA programme (https://www.gov.br/icmbio/pt-br/assuntos/monitoramento), which aims to strengthen the dialogue around environmental issues through the sharing of information and the formulation of questions involving researchers, area managers and communities. A set of procedures has been established to gather data using simple, low-cost techniques, prioritizing the participation of local actors, accompanied by the sharing of analyses and collective interpretation of results. In addition, the multidisciplinary Synergize network (https://synergize.xibe.org/about/) focused on understanding the human-driven and climate-associated disturbances affecting the Brazilian Amazon aquatic and terrestrial biodiversity by gathering community-level datasets on insects, particularly ants, dung beetles [65,66] and aquatic insects [14]. In East Africa, the Kibale National Park in Uganda has documented a decline in arthropod abundance over almost four decades, with a significant decrease in the moderately logged forest area. Various factors, including temperature increase, changes in mammal species, forest structure and landscape alterations outside the park, contribute to the complexity of understanding the drivers of these changes, highlighting the need for long-term, multidisciplinary conservation efforts [67]. However, in other regions, like West Africa and the Indomalaya region, local researchers face obstacles such as the lack of systematic guidelines for monitoring programmes, the absence of taxonomical expertise to create baseline data for local insect checklists, limited access to expensive entomological equipment supplies, and insufficient analytical data and communication skills for publication in scientific journals.

Furthermore, researchers in the Global South express concerns about the logistical complexities of working in remote tropical forests. Some highlight issues related to limited personnel and to local infrastructure, such as inaccessible terrain with no access roads, making it prohibitively expensive to transport researchers and equipment, hindering the establishment and maintenance of long-term monitoring programmes. For example, the probability to do research in the Brazilian Amazon relies heavily on the accessibility and proximity to research facilities [14]. Others point to security risks, such as the fear of local communities in Peru regarding extracting natural resources and limiting access to their terrains. In Colombia, local social unrest and conflicts over illegal crops contribute to challenges in accessing key areas. In countries where conflict zones exist, night-time fieldwork is particularly dangerous, preventing the capture of night insect behaviours, including those of important functional groups like moths.

Besides insect biodiversity monitoring, entomological surveillance significantly contributes to regional public health, particularly in monitoring vector-borne diseases like malaria, dengue, Zika and chikungunya. A study in Rio de Janeiro's Parque Estadual dos Três Picos (PETP) focused on identifying culicids to assess arbovirus circulation risk, revealing vectors such as *Aedes fluviatilis*, *Aedes scapularis*, *Haemagogus leococelaenus* and *Culex* spp. [68]. Continuous surveillance in ecotourism parks is emphasized. In Ghana, another study assessed the efficiency of entomological markers in arbovirus infection risk, highlighting the significance of insect-specific viruses [69]. In Thailand, a study conducted entomological surveillance within 72 h of reported dengue cases, detecting Zika virus (ZIKV) RNA and dengue viruses (DENV-1 and DENV-4) in *Aedes aegypti* samples [70]. Despite advancements in molecular tools for epidemiological monitoring, exemplified in studies like Laroche *et al*. [37], the need for enhanced entomological surveillance persists, especially considering the challenges posed by climate change and global connectivity.

In the realm of biomonitoring programmes for agriculturally significant pest species, substantial investments and funding from multidisciplinary institutions have been made [71]. However, the dissemination of information regarding these practices to local farmers in certain tropical regions appears scattered. A case in point is the Tropical Whitefly Integrated Pest Management (IPM) Project [72], launched in 2007 as a global initiative by research institutions worldwide. This project aimed to tackle the management of whiteflies, including *Trialeurodes vaporariorum*, *Bemisia afer*, and four cryptic species of the *Bemisia tabaci* complex, as pests and vectors of plant viruses in the tropics. In South America, the dynamics of these whiteflies have evolved significantly, with *T. vaporariorum* emerging as a major greenhouse and field crop pest, *B. afer* causing issues in Peruvian lowlands, and *B. tabaci* strains adapting to monocropping practices and pesticide use. The recent invasion of the species *B. tabaci* MED (= Mediterranean) in countries like Argentina, Brazil and Uruguay further adds to the challenges. Molecular analyses have played a crucial role in identifying these species.

However, the implementation of IPM in developing countries encounters challenges owing to confusion about the actual scope of the multifaceted nature of whitefly management. While most strategies underscore the importance of understanding whiteflies' behaviours and preferences, the inclusion of cultural, biological and chemical control strategies can be perplexing for local farmers. Advocating for an integrated approach becomes imperative for effective and sustainable pest control, especially in the face of new challenges posed by invasive species. It is crucial to ensure that efforts focus on mitigating constraints and fostering broader adoption, thereby maximizing positive impacts on sustainable agriculture and pest management [73].

The sustainability of biodiversity, public health surveillance and agricultural biomonitoring programmes hinges not only on sustained funding commitments and active involvement from local communities and stakeholders [10,12,15] but also on the recognition of these challenges by the entire entomological community. This acknowledgement can pave the way for advocacy and support for local researchers grappling with these hurdles.

## 3. Opportunities for advancement

Building on the challenges identified by local researchers in the Global South, we present strategies to enhance monitoring capacity in this region. The initial crucial step is the acknowledgement of our local challenges. When the entomological community comprehends and recognizes the specific requirements for enhancing local monitoring programmes across this region, it sets the stage for robust collaboration and partnerships with local researchers to overcome the challenges.

Now more than ever, engaging local communities and stakeholders in understanding the vital ecosystem services provided by insect and arthropod communities is crucial. In addition, we need to start working on transverse disciplines (e.g. conservation psychology) that can allow us to evaluate human perceptions towards insects and their ecosystem services across diverse group cultures and communities to better build stakeholder and public advocacy conservation strategies for insects [74,75].

As highlighted earlier, many countries in the Global South face challenges in funding science, particularly biodiversity and monitoring programmes, owing to historical or socio-economic factors. While much of the research in these regions has been driven by international collaborations and private academic institutions, the question arises: is this information reaching beyond the scientific community? Citizen science allows the non-scientist public to participate in and contribute to scientific research and brings them closer to science. This contributory model of citizen science brings together scientists and other members of the general populace to collect and process data and to discuss and make meaningful data-driven decisions on threats to biodiversity and the environment [76,77]. This crowdsourcing, contributory model in the research effort, *viz*. ‘public participation in scientific research’ as described in Shirk *et al*. [78], has grown over the years and has proven to be an invaluable collaborative effort among various stakeholders for collecting and tracking large-scale ecological and biodiversity data across multiple geographical regions, which would not have been possible using conventional research methods [79]. Thus, citizen science was used on a large scale to monitor global insect populations, thereby providing a feasible way to address the gap in data for most insect taxa across the world [1,2] for outcomes ranging from research to building community-level capacities for conservation action and policy-making [78]. It should be noted that, although there are a handful of global insect monitoring initiatives currently, such as Global Butterfly Monitoring, Wide Pollinator Monitoring and Global Mosquito Alert [79], there are challenges, such as a disconnect with scientists and a lack of resources in the Global South [80] and many of the well-developed national-level monitoring programmes exist primarily in Europe and North America [79], leading to a taxonomic and geographical bias in data collected [80]. Therefore, there is an urgent need to further develop these programmes with local experts in the Global South. It is noted that platforms like iNaturalist (https://www.inaturalist.org) are widely being used in Latin American countries; however, there is still a lack of integration in Asia and Africa. One of the most significant issues is that these platforms suffer from biased data owing to the heavy contributions from the Global North [81]; facilitating the engagement of local experts on these platforms and integrating them with local initiatives (e.g. Butterflies of India, https://www.ifoundbutterflies.org/) will increase their global impact. Further long-term engagement of these stakeholders is essential to contributing to the meaningful assessments of biodiversity change [79].

While we actively work on building local expertise capacity through citizen science, it is equally important to empower local communities to appreciate their biodiversity, integrate it into their way of life and foster sustainable strategies in agriculture, ecotourism and industrial innovation. Implementing outreach programmes targeting the public through cultural activities can serve as a valuable approach to enhancing public knowledge about the ecosystem services provided by this megadiverse group of insects. Recently in Colombia, non-profit organizations and local governments have been interested in the organization of cultural events that bring citizens together from different cities within the country that highlight insect biodiversity. Here are some examples: Insectopolis [82] is a one-week lecture-based opportunity for non-academics, amateurs and academics to interact around entomological topics and is mainly hosted in public libraries around Cali, Valle del Cauca; Festival ARTropodo is an annual Bug Film Festival to showcase the fascinating and diverse world of insects and other arthropods through the art of cinema, while fostering appreciation and understanding of these often misunderstood creatures (https://www.masuyfundacion.org/en/festivalartropodo); and LepiFEST is an event hosted in Manizales that is trying to bring, through photography, lectures and workshop activities, a better understanding of the ecosystem services that Lepidoptera provide (https://www.inaturalist.org/projects/lepidoptera-colombiana). We hope that the continuation of these outreach and educational programmes will have a positive impact on Colombian insect diversity and engage local stakeholders.

In addition to using festivals to engage with the public, another valuable citizen science collaboration is to involve communities in research processes from the beginning, including designing the project, implementation and dissemination of results through stakeholder engagements at the community level, and educational activities in schools, museums, zoos and other public spaces that promote biodiversity conservation. Moreover, there is a need to provide training and capacity-building opportunities for community members, empowering them to participate in and benefit from research within their communities, both in rural and in urban areas.

Indigenous knowledge systems (IKS) consist of institutionalized traditional and local knowledge and practices existing within and developed by indigenous people and communities from specific regions, that have been passed down through generations [15,83–85]. IKS can provide useful tools and solutions to many conservation and societal problems as these people have lived and continue to live in harmony with nature [84]. For example, in Kenya, farmers from the Makueni District use indigenous knowledge in their practices to adapt to rainfall variability. This knowledge can be used to predict insect emergence patterns and many other life traits and behaviours. In addition to agriculture, these knowledge systems can and have been used in other fields such as biodiversity conservation, interpreting climate change, provision of food sources [84], river, land and general resource management [83], ecosystem preservation, fishing, hunting and medicine [85]. Additionally, in the Neotropical region, there are many indigenous and ethnic territories that can provide important areas for insect conservation [15,86]. Particularly, Indigenous and Community Conservation Areas (ICCA) are very effective in preventing overstepping land expansion in protected areas such as the Brazilian Cerrado [87,88], so actively involving indigenous communities in insect conservation projects can significantly affect declining species.

In order for us to communicate and involve these broad communities it is important to provide outreach and educational tools in the local languages, such as Spanish, Portuguese or French [74,89]. English has for centuries been the dominant language for communicating science [87]; however, it is essential to support local languages, allowing local communities and stakeholders to have this primary information in their local language. Therefore, it is important for entomological journals to allow Global South researchers to publish their contributions in their local language [33]. In addition, the creation of social media platforms and local educational resources promotes the interaction of these discoveries towards a broader audience [88].

Furthermore, collaborations and networking can significantly contribute to technological advancements in monitoring practices [40]. The utilization of molecular biology and barcode genes stands out as an effective approach to delve into the genetic layer of biodiversity [90–92]. While recent progress in environmental DNA (eDNA) monitoring for terrestrial and aquatic arthropods is noteworthy [11,93,94], there are several methodological challenges that researchers in the Global South may encounter. Apart from the financial constraints associated with these technologies, the challenge lies in the substantial difficulty posed by the persistence (decay) of DNA in the varied environments found within the tropics. Additionally, the lack of comprehensive genetic reference data in many of these countries hinders the improvement of taxonomic resolution in studies. Therefore, improving the funding for national biological collections within the countries will be important for constructing the reference data [95]. The vast diversity of species in this ecosystem necessitates further development, as the abundance of species may not be sufficient to trace the entirety of insect diversity during a particular season. Although some of these technologies are beginning to offer more cost-effective versions and methods of implementation, overall their high cost makes it challenging to implement long-term monitoring programmes in most of the Global South [92].

Another future direction can be fostering remote sensing insect detection [96] as recent indirect and direct methods can play pivotal roles, offering complementary insights into the ecological dynamics of insect populations [96]. However, some indirect detection methods delve into the environmental repercussions of insect presence, focusing on signs such as habitat disturbances [97,98]. Spectral feeding signs, discerned through plant indices like normalized difference vegetation index (NDVI) and multispectral satellite data, enable the monitoring of insect-induced plant stress and biomass loss [96]. This approach, especially evident in mapping forest defoliation patterns caused by caterpillars and sawfly larvae, benefits from the high temporal resolution of satellite-derived data [99]. Additionally, structural feeding signs, identified through light detection and ranging (LiDAR) and synthetic aperture radar (SAR) technologies coupled with machine learning, provide a nuanced understanding of changes in vegetation structure resulting from insect feeding [100]. LiDAR excels in mapping defoliation caused by specific insect species, showcasing superior performance when combined with structural information [101]. The integration of radar and optical data further enhances detection probabilities, particularly in regions with persistent cloud cover.

On the other hand, direct detection methods involve a more immediate and targeted approach to studying insect populations [96]. Vertical-looking radar (VLR) systems, capable of autonomously monitoring insect migration patterns, offer detailed information on the shape, mass, altitude and movement of individual flying insects [102,103]. This transformative technology has been instrumental in quantifying seasonal and diurnal patterns of insect migration for various taxa. Entomological LiDAR, with its shorter wavelength compared with microwaves, excels in detecting smaller insects at close range, providing information on position, cross-sectional area and wingbeat frequency [96]. Additionally, passive sensing methods, such as thermal imaging, offer opportunities for detecting insect aggregations through the elevation of collective body temperatures [96]. As advancements continue in indirect and direct detection techniques, a holistic understanding of insect behaviour, abundance and ecological impacts emerges, contributing to more effective conservation and management strategies.

However, we need global building capacity for coding, programming, molecular biology and remote sensing skills to start looking more into providing the best outcomes for the vast diversity of landscapes that are within the tropical regions. We encourage support of local workshops and brainstorming sessions in the Global South; this can be helpful in the implementation of these technologies across the diversity of ecosystems present in the Global South. Beyond financial barriers, the lack of access to underlying design or source code for many of these technologies restricts users' ability to adapt or maintain them independently. It is essential to embrace open access and examine any perceived challenges in adopting practices across the Global South. Open-source hardware and software align with fundamental open science practice fostered by UNESCO's recommendation [104].

Providing and facilitating spaces for Global South researchers to interact with those from developed countries can strengthen local and international networks, prioritizing diverse areas with gaps in entomological knowledge in tropical environments [95]. For instance, the ‘Status of Insects,’ a National Science Foundation (NSF)-funded research coordination network (https://statusofinsects.github.io/index.html), has provided a significant opportunity for entomologists worldwide to connect and collaborate towards advancing data gathering and analysis, and solutions for insect decline. Promoting local in-person meetings to be more inclusive of Global South participation and/or offering hybrid participation options for global meetings can help these countries reduce travel and accommodation costs. Such networking opportunities can foster meaningful collaborations, potentially addressing limited funding opportunities, mitigating article processing fees (including open access) and sharing cost-effective technological advances [95].

Overall, we are hopeful that equitable collaborations across the world will advocate and improve our understanding towards the actual status of insects in the Global South. It is vital to build a diverse and equitable entomological community [105] around the world to preserve our biodiversity and our existence as humankind.

## Data Availability

The data are provided in the electronic supplementary material [106].

## Endnote

1 The 'Global North' and ´Global South́ are often compared, with distinctions typically revolving around differences in wealth, economic development, income distribution, and the level of democracy, as well as political and economic freedom, as measured by various indices. These terms do not refer to geographical position alone. The Global South broadly comprises Africa, Latin America and the Caribbean, Asia (excluding Israel, Japan and South Korea), and Oceania (excluding Australia and New Zealand). The Global North broadly comprises North America and Europe, Israel, Japan, South Korea, Australia and New Zealand.
